# TPdsm: a method based on TabPFN for prediction of deleterious synonymous mutations

**DOI:** 10.1093/bioadv/vbag194

**Published:** 2026-07-13

**Authors:** Bing Zeng, Min Hu, Zhonghui Cui, Siting Zhou, Weiwei Dai

**Affiliations:** Aier Academy of Ophthalmology, Central South University, Changsha, 410004, China; Aier Institute of Digital Ophthalmology & Visual Science, Changsha Aier Eye Hospital, Changsha, 410004, China; Shenzhen Aier Eye Hospital, Aier Eye Hospital, Jinan University, 518000, China; Shenzhen Aier Ophthalmic Technology Institute, Shenzhen, 518000, China; Aier Academy of Ophthalmology, Central South University, Changsha, 410004, China; Aier Institute of Digital Ophthalmology & Visual Science, Changsha Aier Eye Hospital, Changsha, 410004, China; Aier Institute of Digital Ophthalmology & Visual Science, Changsha Aier Eye Hospital, Changsha, 410004, China; Anhui Aier Eye Hospital, Anhui Medical University, Hefei, 230061, China; Aier Academy of Ophthalmology, Central South University, Changsha, 410004, China; Aier Institute of Digital Ophthalmology & Visual Science, Changsha Aier Eye Hospital, Changsha, 410004, China; Aier Academy of Ophthalmology, Central South University, Changsha, 410004, China; Aier Institute of Digital Ophthalmology & Visual Science, Changsha Aier Eye Hospital, Changsha, 410004, China; Anhui Aier Eye Hospital, Anhui Medical University, Hefei, 230061, China

## Abstract

**Motivation:**

Assessing the deleteriousness of synonymous mutations is of considerable importance for understanding human health, and the development of corresponding prediction methods offers a rapid and efficient approach. However, the scarcity of training data remains a major bottleneck in building high-performance predictors for synonymous mutation deleteriousness. Herein, we present TPdsm, a novel method for predicting deleterious synonymous mutations.

**Results:**

TPdsm leverages TabPFN, a tabular foundation model specifically designed for small-sample prediction. The features are retrieved from CDsyn, a comprehensive database dedicated to deleterious synonymous mutation prediction. Our evaluation demonstrates that TPdsm delivers better predictive performance than a set of 14 current state-of-the-art predictors, as evidenced by its results across multiple independent testing datasets and real-world cases.

**Availability and implementation:**

TPdsm is available on GitHub at https://github.com/Project4bz2023/TPdsm. The pre-computed score of TPdsm can be accessed at https://doi.org/10.5281/zenodo.18265619. The result can be queried at https://bingtseng-tpdsm.share.connect.posit.cloud/.

## 1 Introduction

Synonymous variants are a type of coding variant that occurs in bases but doesn’t alter the encoded amino acids. They are second only to missense variants in human genomes (Sharma *et al.* 2019). Although their role in human diseases is unsettled, recent research acknowledges that some synonymous mutations are non-neutral functional mutations, which are validated by experiments (Shen *et al.* 2022, 2024, Niu *et al.* 2026). The pathogenic mechanisms of synonymous mutations include splicing site disruption, mRNA stability alteration, disruption of post-transcriptional m6A modification, translation efficiency dysregulation, protein folding abnormality caused by codon usage bias changes, etc. (Lin *et al.* 2023, Lan *et al.* 2025).

Since experiments are costly and time-consuming to determine the functional impact caused by synonymous mutations, there is a need to develop a prediction tool to distinguish deleterious synonymous mutations from polymorphic synonymous variants.

In fact, several well-known prediction tools for deleterious synonymous mutations have been developed, such as SilVA, DDIG, PrDSM, usDSM, etc. (Buske *et al.* 2013, Livingstone *et al.* 2017, Cheng *et al.* 2020, Tang *et al.* 2021, Lin *et al.* 2023). However, most tools have some limitations (Table S1): (i) Most tools are based on traditional machine learning models, with insufficient generalization ability on the small-sample and high-dimensional synonymous variant datasets; (ii) Many tools rely excessively on existing prediction scores, while population frequencies and novel features related to splicing are less likely to be utilized; (iii) Most tools either provide no precomputed results or only offer precomputed results for the hg19 genome assembly. Thus, the performance and availability of those tools could still be improved (Wang *et al.* 2023).

TabPFN (2.0.9) is a tabular prior-data fitted network that is suitable for small to medium-sized tabular data and yields excellent performance for datasets containing fewer than ten thousand samples (Hollmann *et al.* 2025). Also, TabPFN provides a strategy to extend its application to larger datasets, such as the RF_PFN extension. We used the official universal pre-trained weights of TabPFN and did not apply specific pre-training priors for genomic data in this study. Although the genomic feature exhibit non-linearity and sparsity, TabPFN has advantages in the following aspects: (i) it is pre-trained on one million synthetic tabular classification tasks to learn a universal prior for tabular data distribution (Hollmann *et al.* 2025, Kolberg *et al*. 2025); (ii) The in-context learning mechanism of TabPFN enables dynamic adjustment of its prediction strategy according to the feature distribution of input samples during inference, without the need for retraining (Li *et al.* 2025); (iii) The attention mechanism of TabPFN can automatically focus on the core features critical to the prediction task and mitigate interference from redundant and sparse features; (iv) Designed for small-scale data. Therefore, we decided to utilize the TabPFN to construct a new and efficient model to predict the deleterious synonymous mutations.

In this work, we propose a novel method named TPdsm (TabPFN for prediction of deleterious synonymous mutations). We use CDsyn to annotate the various datasets and obtain the features to train the model and evaluate the performance of the model (Zeng *et al.* 2026). The genetic algorithm (GA) is used to select the high-weighted features (Chu and Beasley 1998), and TabPFN is used to develop the final model (Hollmann *et al.* 2025). TPdsm has four core advantages over mainstream existing tools: (i) we introduce a new algorithm, TabPFN (designed for small-sample prediction), to develop a predictive tool for deleterious synonymous variants. (ii) A GA-based feature selection strategy is used to select crucial features. (iii) We adopt multiple population frequencies and novel splicing features, which are not used in existing models, to achieve accurate modeling of the deleterious effects of synonymous variants. (iv) We provide a pre-computed result of TPdsm on the hg38 version and a user-friendly search website for instant queries. Also, we demonstrate the outstanding performance of TPdsm relative to 14 existing methods in multiple benchmark datasets and real-world datasets.

## 2 Methods

### 2.1 Training datasets and testing datasets

We used the synonymous variants with known pathogenicity in ClinVar (2 02 40 716), HGMD (2 01 602), and dbDSM (1.0) to construct the training dataset. In ClinVar (Landrum *et al.* 2018), the synonymous variants that were labeled with ‘reviewed by expert panel’, ‘criteria provided, multiple submitters, no conflicts’ were selected. Subsequently, the variants recorded as ‘Pathogenic/Likely pathogenic’ were chosen as positive samples, and the variants recorded as ‘Benign/Likely benign’ were recorded as negative samples. In HGMD (Stenson *et al.* 2017), synonymous mutations labeled with ‘DM/DM?’ were selected as positive samples. In dbDSM (Wen *et al*. 2016), we chose 300 synonymous mutations that have been used in many studies as positive samples (Cheng *et al.* 2020, Tang *et al.* 2021, Wang *et al.* 2023). The positive data from three databases comprised the positive data part of the training dataset after removing duplicate data. The positive data and the deduplicated negative data from ClinVar formed the full training dataset. Although several studies used balanced datasets to develop models, we tried to use the unbalanced dataset (Cheng *et al.* 2020, Tang *et al.* 2021, Jin *et al.* 2025). We did this to balance the model’s performance while maximizing the use of negative data. Because the negative data from the full training dataset is relatively large, we tested the fold ratio of benign to pathogenic variants in the range of 0.2–8.6 (TabPFN 2.0.9 supports a maximum input size of 10 000 samples, which restricts the upper threshold of ratio B to 8.6), with a step of 0.2. For the proposed method, we developed the model with a pathogenic-to-benign (PB) ratio of 1:2.8, as it provides the preferred performance (Fig. S1).

We used the two testing datasets from usDSM (Tang *et al.* 2021) as the first testing dataset (usDSM_t1) and the second testing dataset (usDSM_t2). The balanced set from the second testing dataset was used as the third testing set (usDSM_t3). We also used the latest dataset from SynScore for the assessment of TPdsm (SynScore_t1) (Ye *et al.* 2026). Before being used for evaluation, the testing datasets had the overlapping samples with the training dataset removed. The gene sets used in the training and test datasets are listed in Table S2. Table 1 shows the composition of the dataset used in this study. We attempt to verify the model’s performance on population-specific datasets; hence, we constructed four Non-Finnish European (NFE)-specific testing sets by using frequency filtering: gnomad41_exome_AF_nfe > 0, while all other frequencies of gnomad41_exome are equal to 0 or are missing.

**Table 1:** The dataset composition used in this work.

Dataset	Source	Class	Number
**Training set**	ClinVar & HGMD & dbDSM	Positive	1021
		Negative	2858
**usDSM_t1**	PMID: 3,38,66,367	Positive	34
		Negative	2017
**usDSM_t2**	PMID: 3,38,66,367	Positive	25
		Negative	4960
**usDSM_t3**	PMID: 3,38,66,367	Positive	25
		Negative	30
**SynScore_t1**	PMID: 4,14,07,553	Positive	670
		Negative	1295

### 2.2 Feature engineering

The ANNOVAR (2020–06-07) was used to annotate our datasets (Wang *et al*. 2010); the annotation source was from CDsyn (Zeng *et al.* 2026). We utilized 138 features from CDsyn to perform the feature engineering (Table S3). To simplify the operation process, if any sample was not annotated by the CDsyn source, it would be disregarded. For features with missing values, we employed mean value imputation. The only exception was population frequencies, for which we imputed the missing values with zero. Also, we compared K-Nearest Neighbors (KNN, *k* = 5) and Multiple Imputation by Chained Equations (MICE, *n* = 10) with mean imputation to analyse the difference.

TabPFN (2.0.9) was used to build the predictive model because of its advantages mentioned earlier, with the default parameters. Due to the GA having characteristics of randomization, population-based search, and global optimization, it was employed to select significant features for developing prediction tools. The GA configuration was set as follows: Population size = 100, Maximum generations = 100, Crossover rate = 1.0, Mutation rate = 0.1, Elitism count = 5, Convergence threshold = 0.001, AUC threshold = 0.80, Fitness penalty weight = 0.7, Minimum features = 5, Early stopping patience = 10, Cross-validation folds = 5, Frequency threshold = 3, Random seed = 42.

The AUC on the five-fold cross-validation training was used as the fitness function. The fitness of each feature subset was determined by the AUC, with a penalty factor of 0.7 applied to subsets achieving an AUC below 0.80 to maintain population diversity during evolution. We selected 0.80 as the criterion because 0.80 was the common lower threshold for predictive models on clinical datasets (Gunning *et al.* 2021). The GA terminated upon meeting any of the following criteria: reaching the maximum generation limit, early stopping with no fitness improvement for a fixed number of consecutive generations, or convergence when inter-generational improvement was below a minimal threshold. Through this process, we identified 22 features in total, and these features were used to train the model, which were listed in Table S4.

We adopted a strict 5-fold cross-validation framework, where all feature selection and model training were strictly confined within the training folds to avoid data leakage. Furthermore, multiple independent external datasets and real-world cases were used to validate the generalizability of TPdsm and demonstrated its low risk of overfitting.

### 2.3 Assessment of TPdsm performance

To comprehensively analyse the performance of the model built using TabPFN (2.0.9), first, we trained two LightGBM (2.3.1) models with hyperparameter tuning for comparative analysis. One used the same training dataset as TPdsm (PB ratio = 1:2.8), and the other used the full training dataset. Unless otherwise stated, all subsequent training datasets and feature lists align with TPdsm. Second, we compared TPdsm with 14 mainstream predictors in four testing datasets, four real-world cases, and a series of simulated unbalanced testing sets. Third, we compared the computing resources and time consumption in TPdsm and CADD.

Given that LightGBM reportedly performs strongly for imbalanced data, we tested two approaches to evaluate the performance of LightGBM models. Both of two models used Optuna (4.0.0) with a Tree-structured Parzen Estimator sampler and MedianPruner for early termination of unpromising trials. A maximum of 100 trials was conducted. The objective function maximized the Matthews Correlation Coefficient (MCC), computed via 5-fold stratified cross-validation with early stopping (patience = 50 rounds). All trials were parallelized using joblib (n_jobs = −1). The ranges of the hyperparameters were set as follows: n_estimators (100, 1, 000), learning_rate (0.005, 0.3), num_leaves (20, 200), subsample (0.5, 1.0), subsample_freq (1, 10), colsample_bytree (0.5, 1.0), max_bin (200, 300), max_depth (3, 15), reg_alpha (1e-8, 10), reg_lambda (1e-8, 10), min_split_gain (0.0, 1.0), min_child_weight (1e-5, 10), min_child_samples (5, 100), and boosting_type (gbdt, dart, goss). The model trained on the same training dataset as TPdsm is named LightGBM_same_trainset. Another model that used the full training dataset is named LightGBM_full_trainset. The parameters used for these four models are listed in Table S5.

CADD, DANN, DDIG, Eigen, EnDSM, Fathmm_MKL, Fathmm_XF, frDSM, PhD_SNPg, PrDSM, SilVA, Syntool, and usDSM were chosen as a comparison; the computed scores were derived from CDsyn (Zeng *et al.* 2026). SyMetrics was also used as a comparison; the prediction result was obtained based on the official website (Bundalian *et al.* 2026). The thresholds of each tool were from original research or used 0.5 as the default. For TPdsm, we used the threshold calculated by the Youden index to divide the variants into deleterious and benign. Unless otherwise specified, we evaluated model performance using common metrics such as area under the curve (AUC), area under the precision-recall curve (AUPRC), MCC, and F1 score.

For AUC and AUPRC, we computed the 95% confidence intervals (CIs) and the statistical *P*value of pairwise comparison. CIs were computed using adaptive strategies. For AUC, the DeLong method was applied for *n* ≥ 500, BCa bootstrap (5000 resamples) for 50 ≤ *n* < 500, and point estimates only for *n* < 50. For AUPRC, BCa bootstrap was used for imbalanced datasets (positive rate < 0.3 or > 0.7; 2000–5000 resamples based on sample size) or balanced datasets with *n* < 500 (2000–5000 resamples), while percentile bootstrap (1000 resamples) was applied for balanced datasets with *n* ≥ 500. For *n* < 50, only point estimates were reported. Pairwise comparisons against the reference model employed two-sided DeLong tests (*n* ≥ 500) or paired bootstrap tests (*n* < 500), with *P*-value adjusted using the Benjamini-Hochberg FDR procedure.

The deleterious synonymous mutations in Saturation Genome Editing (SGE) were retained to demonstrate the practical utility of TPdsm. We collected the 76 and 15 deleterious synonymous variants of *BAP1* and *RAD51C* (Olvera-León *et al.* 2024, Waters *et al.* 2024), respectively, following the method recorded in SyMetrics (Bundalian *et al.* 2026). The true positive count was utilized to assess the performance of models, because only experiment-verified deleterious variants were chosen. Two clinical case studies on the ABCA4 gene and the AVPR2 gene were used to prove the superiority of TPdsm (Kaltak *et al.* 2023, Bundalian *et al.* 2026). In these cases, we employed the summary of the true positive count and the true negative count to evaluate the model’s performance. Additionally, to assess the model’s performance on imbalanced samples, we combined the four testing datasets to generate simulated test sets with various PB ratios, ranging from 1:1 to 1:1000. For TPdsm, we split the simulated test sets into multiple small subsets (≦10 000) as needed to meet the requirements of TabPFN (2.0.9).

We compared the computing resources and time consumption on TPdsm and CADD (https://github.com/kircherlab/CADD-scripts). We did not use other tools such as usDSM and enDSM because they are unavailable. We used 1000, 10 000, 100 000 and 1 000 000 VCF files built from the CDsyn database to perform the assessment.

### 2.4 Access to pre‑calculated TPdsm score

To facilitate the usage of TPdsm, not only did we provide the pre-calculated score on Zenodo (https://doi.org/10.5281/zenodo.18265619), but we also constructed a website for online query (https://bingtseng-tpdsm.share.connect.posit.cloud/) using the R package Shiny (1.10.0). TPdsm was available on GitHub at https://github.com/Project4bz2023/TPdsm. TPdsm must use features that follow the order we provide. Otherwise, TPdsm will not work correctly. The recommended configuration is CPU (8 cores, 2.5 GHz or higher), system memory (32 GB or higher), and GPU (NVIDIA discrete GPU with 8 GB+ video memory, 1.9 GHz or higher boost clock).

## 3 Results

**Figure 1:**
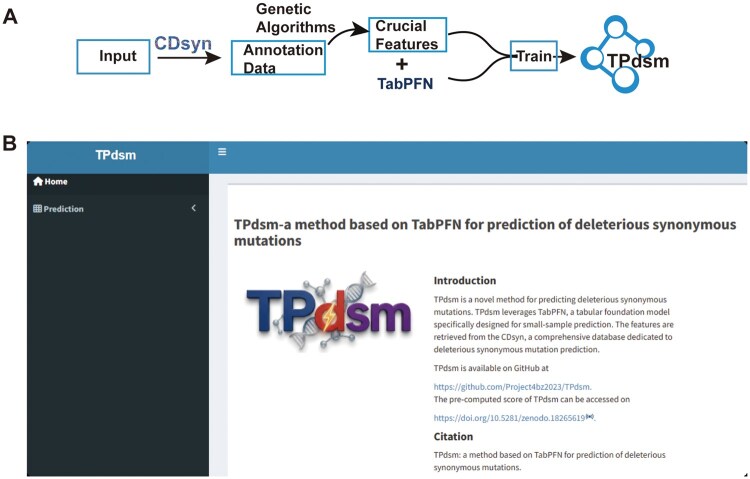
TPdsm is a novel predictor for deleterious synonymous mutations. (A) The workflow of developing TPdsm. (B) The online website for querying the TPdsm score.

We combined the GA and TabPFN to construct a novel model, TPdsm, for predicting deleterious synonymous mutations (Fig. 1A). We demonstrated that the predictor based on TabPFN has superior capacity, and feature engineering following the GA can improve the performance of the model. Additionally, we provided a pre-computed score on Zenodo and built an online website for instant queries (Fig. 1B).

### 3.1 The crucial features used in TPdsm

The features used in TPdsm can be seen in Table S4. Four testing datasets were utilized to demonstrate the outstanding performance of TPdsm. The SHAP value of the features used in TPdsm can be seen in Fig. S2. Of the 22 features, we introduce three novel ones: syntool_rankscore, delta_psi_max, and population frequencies-related features, including gnomad41_exome_faf99, gnomad41_exome_faf99_max and gnomad41_exome_AF_eas. Among them, the CADD_PHRED score contributes the most, followed by the SilVA score and then the delta_score from SpliceAI (Jaganathan *et al.* 2019). Surprisingly, gnomad41_exome_faf99, the population frequency, ranks fifth in the contributions, which is rarely mentioned in the existing studies (Buske *et al.* 2013, Cheng *et al.* 2020, Tang *et al.* 2021). It may imply the significance of population frequencies in the development of the prediction model for deleterious synonymous mutations. Although gnomad41_exome_faf99 has a relatively high contribution in the SHAP analysis, we speculate that it didn’t reflect a bias in the training data; it is more prone to biological selective pressure. In Table S6, we can see that the proportion of benign variants is relatively uniform across the two classes of variants (gnomad41_exome_faf99 ≧ 0.05 & gnomad41_exome_faf99 < 0.05). Additionally, we analysed the generalizability of the population frequencies feature across different ethnic backgrounds. We examined the performance of TPdsm on the four NFE-specific testing sets. The results (Table S7) demonstrate that TPdsm still delivers the same performance in NFE-specific datasets.

### 3.2 The effect of the training dataset with different PB ratios on the model’s performance

To explore how to maximize the utilization of the negative data from ClinVar, we analysed how training datasets with different PB ratios affected the model’s performance. As we can see from Fig. S1, when the PB ratio is 1:2.8, the mean AUC value of the four testing datasets is the highest. Notably, the mean AUC value of the PB ratio of 1:2.8 is significantly higher than that of the ratio of 1:1. We therefore adopted the training dataset with a 1:2.8 ratio to construct the models.

### 3.3 The effect of different imputation methods on the model’s performance

To analyse the effect of different imputation methods on a model’s performance, we tried two other imputation methods, KNN and MICE. As shown in Table S8, we can see that regardless of the imputation method chosen, the model built with TabPFN remains superior to the compared model across three of four test sets. Although switching missing data imputation methods exerted a relatively minor effect on model performance, we recommend reselecting features and retraining models for the other two strategies. This ensures an objective reflection of their actual performance.

### 3.4 Comparison of TPdsm and other models built with various methods

To explore the potential of LightGBM, we conducted two additional analyses using LightGBM with Optuna. One used the same training set as TPdsm (PB ration = 1:2.8), while the other used the full dataset. As presented in Table S9, LightGBM, which used the same training dataset as TPdsm (LightGBM_same_trainset), exhibits a higher mean AUC value than models using the full training dataset in the testing sets (LightGBM_full_trainset). More importantly, TPdsm was superior to LightGBM_same_trainset in AUC across all testing datasets. Combining the results of the two comparisons, although LightGBM-based models had higher metrics on one or two test sets, the difference values were small. Thus, we speculated that TabPFN was the preferred approach in developing new tools. The reason was likely that universal prior knowledge brought by pre-training enables TabPFN to have stronger generalization ability and non-linear fitting ability on small-sample, high-heterogeneity genomic data.

### 3.5 The performance of TPdsm in multiple testing sets and real-world cases

**Figure 2:**
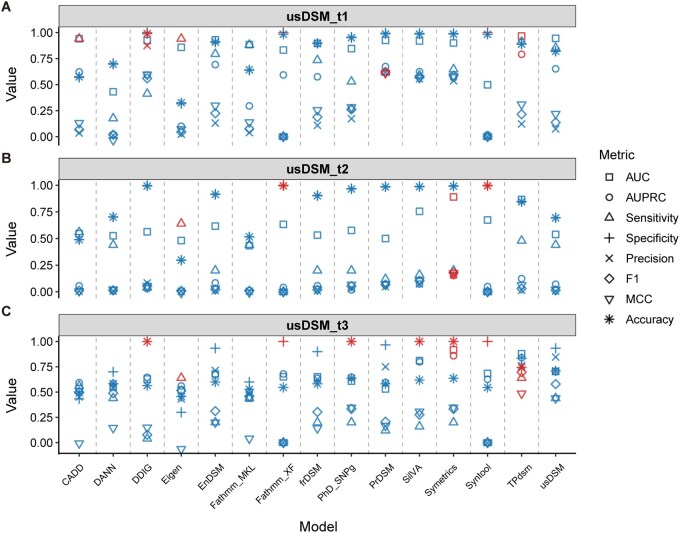
The performance of TPdsm and 14 tools in three testing datasets. Each graph corresponds to a separate testing dataset. (A). The usDSM_t1; (B). The usDSM_t2; (C). The usDSM_t3. The different symbols represent different metrics used to evaluate the performance. The symbol colored red means the model performs best in the metric.

In the evaluation on the first testing dataset (Figs. 2A, S3A and S4A), TPdsm outperforms usDSM in all metrics, though this comparison lacks statistical power. Its AUC was more than two percentage points higher, and AUPRC is approximately fourteen percentage points higher. The latter is the best tool except for TPdsm. When the benchmark dataset changes to the second testing dataset (Figs. 2B, S3B and S4B), although SyMetrics shows superior metrics compared to TPdsm, the difference between them is not statistically significant. A considerable margin in AUC is observed between the proposed method and the third-best performer, with differences of about eleven percentage points. Besides, the trends remain the same for AUPRC. Notably, all models show a mediocre AUPRC in usDSM_t2.

We therefore speculate that this is a common issue with imbalanced data, representing a promising direction for improving predictive model performance. In the third testing dataset (Figs. 2C, S3C and S4C), SyMetrics also exhibits higher values than TPdsm on four metrics without a statistical difference. Excluding SyMetrics, the TPdsm still maintains high performance and performs well in six out of eight metrics, outperforming the third-best approach by six percentage points in AUC and four percentage points in AUPRC. While other models perform better across several metrics, we can improve this situation through threshold optimization.

Although SyMetrics outperforms TPdsm on the usDSM_t2 and usDSM_t3 datasets, no statistically significant differences exist between the two approaches. Furthermore, usDSM_t3 is essentially derived from usDSM_t2 (see “Methods” section). Therefore, the observed performance gaps between these two datasets should be interpreted with caution. Correspondingly, despite TPdsm exhibiting superior performance over all remaining models, no statistically significant disparities are detected when partially comparing its metrics with CADD, DDIG, EnDSM, frDSM, Syntool, PhD_SNPg, and SilVA. On the usDSM_t1 dataset, TPdsm significantly surpasses SyMetrics, yet its comparative outcomes against CADD, PrDSM and usDSM also require prudent interpretation.

**Figure 3:**
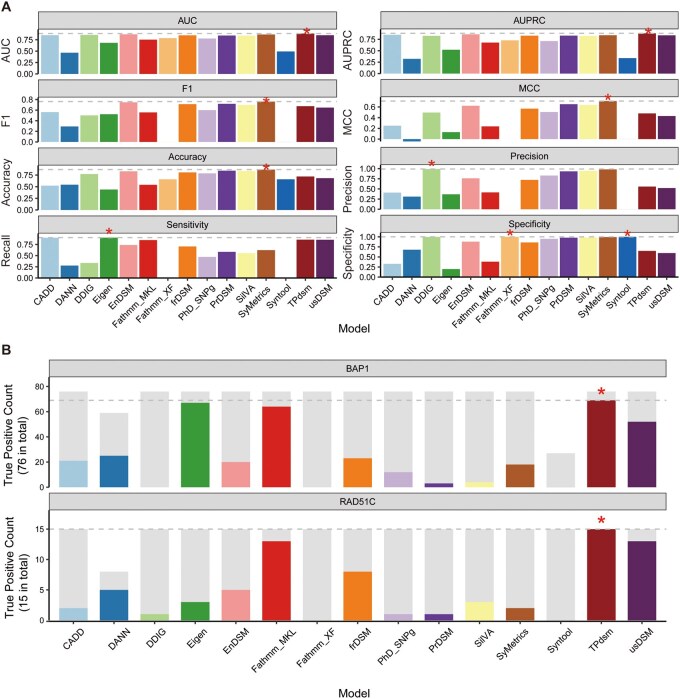
The performance of TPdsm and 14 tools in an independent testing dataset (SynScore_t1) and two real-world cases. (A) The performance of TPdsm and other tools in the independent testing dataset (SynScore_t1) was assessed in eight metrics; each graph corresponds to a separate metric. The bar labeled with a red star means the model performs best in the metric. (B) The performance of TPdsm and other tools in the two real-world cases. The upper graph is for *BAP1*, and the lower graph is for *RAD51C*. The height of gray bars denotes the total number of predictions generated by the model in the evaluation, and the height of bars in other colors indicates the number of correctly predicted results by the model in the evaluation. The bar labeled with a red star means the model performs best in the comparison. The gray dashed line marks the position of the best performance score.

To further assess the performance of TPdsm, we used more datasets. Firstly, we collected another independent testing dataset published recently (Ye *et al.* 2026). TPdsm has the highest AUC and AUPRC on this test (Figs. 3A, S3D and S4D). In the two real-world cases of *BAP1* and *RAD51C* (Fig. 3B), TPdsm identifies the largest true positive variants, 69/76 in *BAP1* and 15/15 in *RAD51C*. Fathmm_MKL and usDSM exhibit second-best performance in *RAD51C*, and Eigen shows inferior performance to TPdsm in *BAP1*. SyMetrics performs relatively poorly on these two datasets. However, some models, such as Fathmm_XF and Syntools, failed to generate predictions for the entire dataset. In brief, TPdsm, built in TabPFN, shows excellent performance in multiple test sets.

In the two additional case studies of *ABCA4* and *AVPR2* (Table S10), SyMetrics is the best-performing model across all models; TPdsm follows it. Eigen exhibits comparable performance with TPdsm for *ABCA4*, yet TPdsm still achieves a higher Total_Count value than Eigen for *AVPR2*. Finally, we made a summary of the performance ranking table of TPdsm and 14 existing tools on all test sets and real-world cases (Table S11). Although SyMetrics outperforms TPdsm on a few test sets, TPdsm claims the top spot across all evaluations. This clearly shows that TPdsm is overall superior to existing methods.

### 3.6 The performance of TPdsm in testing sets with various PB ratios

To further research TPdsm’s performance on imbalanced samples, we evaluated TPdsm on testing sets with various PB ratios. As shown in Table S12, as the imbalance of the PB ratio increases, the Precision, MCC, and F1-score of TPdsm decrease to varying degrees (with the PB ratio varying from 1:1 to 1:1000). This is a common phenomenon in extremely imbalanced classification tasks. The exponential increase in the negative samples leads to a significant increase in false positive results, which in turn lowers precision. However, on all test sets with different imbalance ratios, the AUC, sensitivity, accuracy, specificity and AUPRC remain relatively stable, especially compared to other metrics. For the problem of acceptable sensitivity but low precision, we advise the user to flexibly adjust the classification threshold according to their own application scenarios.

### 3.7 The computing resources and time consumption in TPdsm and CADD

To compare the computational time and memory usage between TPdsm and CADD, we used multiple VCF files as testing files on the same hardware. Table S13 clearly shows that TPdsm runs faster than CADD across all tested VCF files, though it consumes far more resources. TPdsm has stable GPU usage at 3.2GB, while CADD requires no GPU memory. Additionally, TPdsm’s CPU usage is 40 to 100 times that of CADD. Therefore, when computational resources are relatively abundant and operational speed is prioritized, using TPdsm is the preferred choice.

### 3.8 Limitations and future perspectives

Although TPdsm introduces a pioneering framework for predicting deleterious synonymous mutations by utilizing a deep learning-based Transformer model, we acknowledge that applying TabPFN to genomic data requires more exploration and further validation. However, there are still some limitations in our study. First, the size of the four testing datasets, especially the third one, is relatively small, which may introduce potential bias into the evaluation results. Second, the utilization of GA may result in diminished interpretability of the model. Third, relying solely on existing features might constrain the model’s performance. Also, utilizing the scores from predictors, especially from competing predictors, may compromise the objective assessment of the TPdsm’s performance. Since the impact of PBratio on model performance has not been thoroughly assessed in this study, our results may be subject to limitations. Future work should focus on improvements in the above aspects to further enhance the model’s capabilities. With the release of pre-computed scores of TPdsm and a query website, we expect TPdsm to be a valuable tool for synonymous mutations and to promote research in human health.

## Supplementary Material

vbag194_Supplementary_Data

## Data Availability

The model, code and datasets are available on *GitHub* at https://github.com/Project4bz2023/TPdsm; the pre-computed score of TPdsm can be accessed on https://doi.org/10.5281/zenodo.18265619. The pre-computed result can also be queried on https://bingtseng-tpdsm.share.connect.posit.cloud/. The annotation file of *CDsyn* can be accessed at https://doi.org/10.5281/zenodo.15226837.
