# Supplementary material for: TPdsm: a method based on TabPFN for prediction of deleterious synonymous mutations
Source: Bioinform Adv. 2026 Jul 13;6(1):vbag194. doi: 10.1093/bioadv/vbag194 (PMC13401442; doi:10.1093/bioadv/vbag194)
Supplement: vbag194_Supplementary_Data [file vbag194_supplementary_data.zip › Supplementary_Data.zip!/Supplementary material.docx]

**Figure S1.** The effect of the training dataset with different PB ratios on a model's performance.

**Figure S2**. The SHAP value of the features in the TPdsm.

**Figure S3**. The AUC curve of TPdsm on four testing datasets. (A) usDSM_t1; (B) usDSM_t2; (C) usDSM_t3; (D) SynScore_t1.

**Figure S4**. The PRC curve of TPdsm on four testing datasets. (A) usDSM_t1; (B) usDSM_t2; (C) usDSM_t3; (D) SynScore_t1.
